# Characterisation of the nociceptive phenotype of suppressible galanin overexpressing transgenic mice

**DOI:** 10.1186/1744-8069-6-67

**Published:** 2010-10-21

**Authors:** Robert JP Pope, Fiona E Holmes, Niall C Kerr, David Wynick

**Affiliations:** 1 Department of Physiology and Pharmacology and Clinical Sciences at South Bristol, School of Medical Sciences, University Walk, University of Bristol, Clifton, Bristol, BS8 1TD, UK

## Abstract

The neuropeptide galanin is widely expressed in both the central and peripheral nervous systems and is involved in many diverse biological functions. There is a substantial data set that demonstrates galanin is upregulated after injury in the DRG, spinal cord and in many brain regions where it plays a predominantly antinociceptive role in addition to being neuroprotective and pro-regenerative. To further characterise the role of galanin following nerve injury, a novel transgenic line was created using the binary transgenic tet-off system, to overexpress galanin in galaninergic tissue in a suppressible manner. The double transgenic mice express significantly more galanin in the DRG one week after sciatic nerve section (axotomy) compared to WT mice and this overexpression is suppressible upon administration of doxycycline. Phenotypic analysis revealed markedly attenuated allodynia when galanin is overexpressed and an increase in allodynia following galanin suppression. This novel transgenic line demonstrates that whether galanin expression is increased at the time of nerve injury or only after allodynia is established, the neuropeptide is able to reduce neuropathic pain behaviour. These new findings imply that administration of a galanin agonist to patients with established allodynia would be an effective treatment for neuropathic pain.

## Findings

The 29 (30 in human) amino acid neuropeptide galanin has a wide distribution in both the peripheral and central nervous systems. The galanin peptide shares homology with only two other known peptides, galanin-like peptide (GALP) [1] and the GALP splice variant alarin [2]. Galanin is expressed at low levels in ~5% of small diameter C-fibre neurons in the intact adult rodent dorsal root ganglia (DRG) [3-5]. Higher levels of the peptide are detected in the primary afferent terminals of the spinal cord (lamina II), the dorsal horn inter-neurons [6], and in a number of brain regions known to modulate nociception, including the arcuate nucleus and periaqueductal grey [7,8]. Galanin levels are strongly and persistently increased in the DRG and dorsal horn of the spinal cord as well as in many regions of the central nervous system following nerve injury. Axotomy of motor nerves elevates galanin mRNA levels by 6-10 fold in the dorsal motor nucleus of the vagus and nucleus ambiguus [9] and a similar increase is found in the facial nucleus following facial nerve axotomy [10]. Galanin levels in the central nervous system are also upregulated in many disease states and in rodent models of these pathological conditions, including Alzheimer's disease [11], stroke induced ischemia [11,12] and in multiple sclerosis [13]. However, the most potent upregulation occurs in the DRG following peripheral nerve axotomy when galanin is rapidly up-regulated by up to 120-fold and is expressed in 40-50% of sensory neurons [14]. A much smaller increase in galanin expression is observed in the dorsal horn after peripheral nerve injury [15] implying that much of the neuropeptide is anterogradely transported to the site of injury. Following the description of galanin expression increasing after nerve injury, the role of galanin in nociception and neuropathic pain has been the subject of a substantial body of research. Studies of intact and nerve injured animals have described a bell-shaped response curve after intrathecal infusion of galanin and galanin agonists [16-18] with inhibition of nociceptive responses at high doses [19,20]. To further define the role played by galanin in nociception, we have previously generated and characterised two transgenic mouse lines that either overexpress galanin in the DRG after nerve injury [21] or constitutively and ectopically in both the intact DRG and after nerve injury [22]. Both lines demonstrate a marked reduction in mechanical allodynia in the spared nerve injury (SNI) model of neuropathic pain [22]. Subsequently, another galanin over-expressing mouse line that ectopically over-express galanin in the DRG under the control of the dopamine beta-hydroxylase promoter has shown a similar decrease in neuropathic pain-like behaviour [23]. Whilst the study of these existing transgenic lines have generated valuable data, they all overexpress galanin embryonically and throughout the animals life. It is therefore desirable to reversibly overexpress galanin in a temporally controllable fashion allowing one to study whether galanin can reverse established allodynia, thus validating galanin as a target for drug discovery.

**Figure 1:**
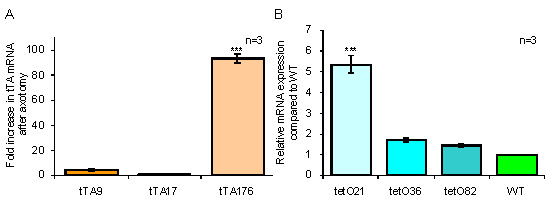
**Quantitative real-time PCR analysis of tTA and tetO lines**. Real-time quantitative PCR was used to measure: A, the fold increase in the level of tTA mRNA in pooled DRG samples one week after axotomy. Line 176 has significantly higher levels of tTA mRNA than tTA9 and tTA17 which do not differ significantly from each other or WT mice. B, expression of total galanin mRNA in pooled DRG samples from intact transgenic and WT mice were compared. Line tetO21, tetO36 and tetO82 were increased by 5.36, 1.69 and 1.44-fold respectively. Line tetO21 was significantly different to the other 2 lines (1-way ANOVA, *** denotes P < 0.001).

To further define the role of galanin in the nervous system after nerve injury, we have used the tet-off system to develop a binary galanin overexpressing mouse line in which the overexpression can be suppressed by administration of the tetracycline analogue doxycycline (dox). Two transgenes were generated both utilising previously described constructs from the murine galanin gene. In order to direct galanin overexpression solely to galaninergic tissue, the previously characterised 20 kb galanin enhancer [21,24] was used to drive tetracycline controlled transactivator (tTA) expression (termed tTA transgene). In parallel, the 4.6 kb galanin genomic locus, containing all 6 exons which has previously been shown to direct high levels of galanin in a number of transgenic lines [22], was inserted downstream of the tetracycline operator (tetO) sequences (termed tetO transgene). Three founder lines were characterised for each transgene. The increase in tTA mRNA expression was measured in the DRG after axotomy of the sciatic nerve in each of the tTA lines using quantitative real-time PCR (Figure 1A). Line tTA176 had a significant increase in mRNA levels after axotomy. The tetO lines were assessed for the level of basal expression in the absence of the transactivator protein, so called "leakiness" (Figure 1B). Quantitative real-time PCR revealed that line tetO21 had a significant degree of basal expression. The tTA line with the greatest axotomy response (line 176) and the tetO line that displayed the lowest level of basal expression (line 82) were subsequently crossed to each other to generate a double heterozygous transgenic line.

**Figure 2:**
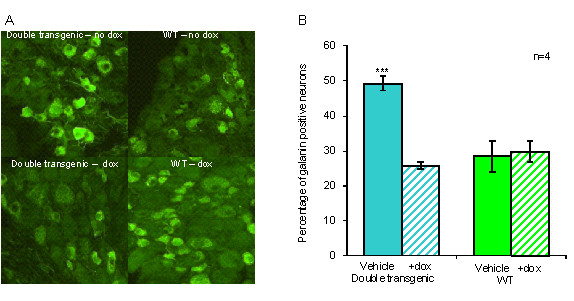
**Percentage of galanin positive DRG neurons in double transgenic lines after axotomy**. A, Representative DRG sections immunohistochemically stained for galanin one week after sciatic nerve axotomy from double transgenic and WT control. Animals were given water (including 5% w/v sucrose) (top panel) or dox (including 5% w/v sucrose) from the time of axotomy until sacrifice (lower panel). B, The percentage of galanin positive neurons in sections from axotomised L4 and L5 DRG with dox at 200 mg/ml (hatched bar) and without dox (solid bar). Double transgenic DRG have a significant overexpression of galanin after axotomy when compared to WT control animals in the absence of dox. No significant differences between transgenic and WT mice were observed when dox was administered (1-way ANOVA *** denotes P < 0.001).

**Figure 3:**
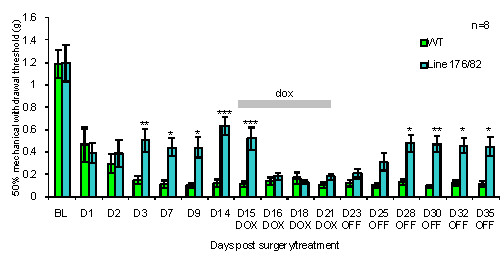
**Mechanical withdrawal threshold responses of double transgenic and WT mice after SNI model of neuropathic pain**. Baseline (BL) recordings were taken before SNI. After surgery, the animals received no dox for 14 days, then dox for 7 days followed by a further 14 days with no dox. Pre-surgery thresholds were not significantly different between genotypes. The WT group developed robust allodynia and this was not affected by the administration of dox. Double transgenic mice developed significantly less allodynia than WT controls between days 3 and 14 of the first "off dox" period. No significant difference between the genotypes was observed from day 2 to 7 of the "on dox" period but a significant difference was again observed by day 7 of the second "off dox" period (2-way ANOVA. * denotes P < 0.05, ** denotes P < 0.01 and *** denotes P < 0.001).

The level of galanin overexpression in the double heterozygous transgenic was assessed at the protein level by measuring the percentage of galanin positive neurons in the DRG one week after axotomy, both in the presence and absence of dox (Figure 2). An approximate 50% increase was observed in the number of galanin positive neurons in the DRG of the double transgenic line compared to WT animals. This overexpression was abolished by administering 200 mg/ml of dox in the drinking water from the time of sciatic nerve axotomy until the animals were sacrificed one week later. Dox administration had no effect on the levels of galanin protein in WT animals. To assess the functional activity of transgene-derived galanin protein, mechanical withdrawal thresholds were measured following the SNI model of neuropathic pain in adult double heterozygous transgenic and WT mice (Figure 3). Mechanosensory thresholds (using von Frey hairs) were measured first in intact animals and then following SNI over a 35 day period consisting of 14 days with no dox, then 7 days on dox, followed by a further 14 days with no dox. The comparison of the double transgenic to WT mice revealed significant differences in the levels of allodynia over the course of the experiment. Allodynia was significantly attenuated after SNI in the double heterozygous transgenic in the absence of dox when compared to WT controls from day 3 to day 14. This finding is consistent with the previously described galanin overexpressing mice, which used the same 20 kb and 4.6 kb constructs used in this study [22]. The withdrawal thresholds of the double transgenic were not significantly different to WT animals following the administration of dox, as the mice became more allodynic suggesting that dox successfully suppresses transgene-derived galanin. Following the withdrawal of dox, there was an increase in withdrawal thresholds over the next seven days, presumably due to the animals metabolising and clearing the dox thus allowing the levels of transgene-derived galanin to rise once again.

The generation of the double transgenic mice address one of the fundamental drawbacks of conventional transgenic overexpression, that of not being able to separate and independently study the role of a gene during development from its role in the adult. Having reversible and temporal control over gene expression allows the researcher much greater flexibility to study the various roles played by a protein in the adult, as compared to the study of animals that overexpress during embryogenesis and throughout the entire life of that animal. These findings demonstrate that the double transgenic line overexpresses galanin in the DRG after sciatic nerve injury and that the overexpression is abolished by dox administration. Further, the level of overexpression is sufficient to significantly attenuate allodynia following SNI. These mice also present a means to potentially rescue the developmental DRG nociceptor losses described in the galanin knockout and thus potentially allow, for the first time, the characterisation of a developmentally normal adult that is null for galanin. Furthermore, the finding that galanin can significantly attenuate established allodynia strongly supports galanin and its receptors as targets for drug development.

## Materials and methods

### Generation of mice

Transgenic mice (CBA/B6 strain) were generated that expressed a) tTA (pTet-tTak - Invitrogen, Paisley, UK) under the control of the previously characterised 20 kb galanin enhancer region [21,24] and b) the 4.6 kb galanin genomic locus [22] under the control of the tetO sequences. Founder mice were initially identified by southern blot using 1.2 kb NcoI-XhoI and 1.9 kb EcoRI-BamHI probes, both probes being excised form the galanin genomic locus, to identify tTA and tetO positive founder mice respectively. Genomic DNA was digested with EcoRI (tTA) and EcoRI + XhoI (tetO). Subsequent genotyping was carried out by PCR using primers 5'-TTTTGACCTCCATAGAAGACACC-3' and 5'-ATGGTAGCGTCAGACGTCCG-3' to detect tetO and primers 5'-GAGTATGGTGCCTATCTAACATCT-3' and 5'-GACTGTGGGTGATCCTCTCC-3' to detect tTA. A band from the endogenous galanin gene is also amplified by these primers as an internal control.

The double transgenic mice were generated by crossing line tetO176 and line tTA82 line. In all experiments, double heterozygote transgenic and WT animals that were age, sex and strain matched were compared and tested with the genotype blind to the experimenter. All surgery and procedures were carried out in accordance with the United Kingdom Animal Scientific Procedures Act 1986.

### RT-PCR and Quantitative real-time PCR

Total RNA extraction, DNAse treatment and re-extraction and reverse transcription are as previously described [25]. Quantitative real-time PCR was performed as previously detailed [26]. The forward primer, reverse primer and internal non-extendable fluorescent probe for tetO and tTA mRNA are detailed below. tetO forward 5'-ACGCTGTTTTGACCTCCATAGAA-3', reverse 5'-TGGCGGGCTGGATGGT-3', probe 5'-CGGGACCGATCCAGCCTCCG-3'. tTA forward 5'-GATAAAAGTAAAGTGATTAACAGCGCATT-3', reverse 5'-CTAGCTTCTGGGCGAGTTTACG-3', probe 5'-ACCTTCGATTCCGACCTCATTAAGCAGCT-3'.

### Mouse surgery, immunohistochemistry and behavioural analysis

Sciatic nerve axotomy, SNI, measurement of mechanical withdrawal thresholds and immunohistochemistry are as previously described [22,24,27]. Where appropriate, animals (WT and transgenics) were given 200 mg/ml of dox (Sigma) in the drinking water. 5% (w/v) sucrose was included in all drinking water to mask the bitter taste of the antibiotic if present.

## Competing interests

The authors declare that they have no competing interests.

## Authors' contributions

DW conceived and designed the study. RJPPP generated the data with the assistance of NCK (molecular biological techniques) and FEH (behavioral analysis). Manuscript was written by RJPP and DW and the final manuscript was read and approved by all authors.
